# The COVID-19 Pandemic in the Nawalparasi District of Nepal: a mixed methods assessment of increased alcohol use and violence against women

**DOI:** 10.1186/s12889-023-14997-1

**Published:** 2023-03-18

**Authors:** Alia Cornell, Ashley Mitchell, Mahesh Puri, Nadia Diamond-Smith

**Affiliations:** 1 Undergraduate Student, University of California Los Angeles, Los Angeles, USA; 2 Doctoral Student, Institute for Global Health Sciences, University of California, San Francisco, USA; 3 Director of Research, Center for Research On Environment Health and Population Activities (CREHPA), Kathmandu, Nepal; 4 Department of Epidemiology and Biostatistics and Institute for Global Health Sciences, University of California, San Francisco, USA

**Keywords:** COVID-19, IPV, VAW, Alcohol use, Households, Economic insecurity, Intervention, SDGs

## Abstract

**Background:**

In Nepal and across the globe, the COVID-19 pandemic has primed an environment for increased rates of violence against women (VAW). This paper explores pandemic-driven economic insecurity and increased alcohol use as instigators of VAW and Intimate Partner Violence (IPV) within newly married households in the rural, Nawalparasi region of Nepal.

**Methods:**

This study is a secondary analysis of data obtained from the *Sumadhur* Intervention pilot study that has been previously described and demonstrates successful implementation of group-based, household-level intervention for women’s empowerment and sexual and reproductive health education (1). Our three sets of data were collected before and during the COVID-19 pandemic. The first set is from a Longitudinal Cohort of 200 newly married women who were surveyed twice a year from February 2017 through July 2020. The second data set is a subset cohort of newly married women, their husbands, and their mothers-in-law (31 women, 31 husbands and 31 mothers-in-law) who participated in *Sumadhur* in January 2021. The third data set was obtained through in-depth interviews in July 2021 from 15 households following *Sumadhur*. The interviews were thematically coded, and subthemes were identified. A t-test of the January 2021 survey data set was run to look at correlations between income loss, alcohol consumption and experience of IPV among newly married women. All other survey data was analyzed for change over time.

**Results:**

At three months after the onset of the pandemic (July 2020), the Longitudinal Cohort survey data from newly married women reported increased rates of husbands’ alcohol use as well as personal experiences of IPV as compared to pre-pandemic averages. There was a statistically significant difference (p < 0.001) in the effects of income loss on increased alcohol use and experience of IPV. Qualitative results iterated the common theme of alcohol use and economic insecurity as upstream instigators of VAW in the community.

**Conclusions:**

In the Nawalparasi district of Nepal, the pandemic has led to unstable economic situations that have instigated alcohol use among men, and increased rates of IPV among young, newly married women, and reports of VAW in the community. We have demonstrated a need for urgent programmatic and policy responses aimed at reducing VAW and IPV and protecting women during times of uncertainty and crisis.

## Background

Over the past two years, the COVID-19 pandemic has created increased stressors on socioeconomic, political, and healthcare systems across the world [1]. These challenges and instabilities have brought about coping strategies, such as alcohol misuse, that have heightened the opportunity for violence [2]. Vulnerable groups, especially women and girls, are most likely to bear the harmful ripple effects of maladapted coping strategies, increasing their risk of experiencing violence [3].

Gender-based violence (GBV) is an act of violence, threat, or coercion that disproportionately affects persons of a particular gender [4]. GBV is rooted in unequal power dynamics between genders that come from larger social constructions and norms of gender. Specifically, Violence Against Women (VAW) is a subset of GBV that results in the harm or suffering of women [5]. Intimate Partner Violence (IPV) refers to acts of physical violence, sexual violence, emotional or psychological abuse and controlling behaviors perpetrated by a current or former intimate partner [6]. In 2022, it was estimated that 27% of women and girls above the age of 15 globally have experienced some form of IPV, although in certain regions and countries, rates are higher [7]. South Asia has one of the highest rates of IPV in the world, with an estimate of 42% of ever partnered women reporting IPV [8]. VAW and IPV remain among the most pressing global health concerns and are a violation of women’s human rights.

Prevention of VAW and IPV is encompassed in Goal 5 and Goal 16 of the United Nations (UN) 2030 Sustainable Development Goals (SDGs) which were initiated in 2015 after the UN Conference on Sustainable Development in Rio de Janeiro, Brazil [9]. Goal 5 is “to achieve gender equality and to empower all women and girls”, which includes eliminating all forms of violence against women and girls and places an emphasis on the importance of adopting and strengthening policies and legislation to protect them [10]. Goal 16 calls for promotion of peaceful and inclusive societies, to significantly reduce all forms of violence and to spread equal access to justice for all [10]. Combined, the 17 Goals emphasize the interconnectedness of women’s safety and empowerment within sustainable progress and building a healthier and more prosperous world. The Government of Nepal submitted Voluntary National Reviews (VNRs) in 2017 and in 2020 to the UN to report on their country’s progress of the goals [11]. These reports detail data collection strategies, budget allocations, partnerships and stakeholders to progress on gender equality in all sectors of society, VAW prevention and the promotion of peaceful and resilient societies [11].

The Government of Nepal has shown commitment to decreasing the prevalence of VAW, and IPV specifically. Since 2009, several laws and plans of action have been initiated with this goal, including the Domestic Violence (Offence and Punishment) Act in 2009 and the National Plan of Action against gender-based violence (in 2010, and from 2013–2018). Furthermore, the new 2015 Constitution of Nepal represents a significant milestone for gender equity and social inclusion, protecting some rights for women, the poor, GBV survivors and other vulnerable and marginalized groups. On August 24^th^, 2018, the House of Representatives passed a four-point resolution on ending VAW and created a high-level mechanism for ensuring its implementation. However, a study mapping institutional, legal and policy responses for addressing GBV revealed many challenges that remain, particularly in enforcing the laws and implementing the policies and programs. Even amidst an adaptive political scene, studies have shown that VAW and IPV are still widespread in Nepal [12]. Women in Nepal are more likely than global averages to experience violence in their households, with estimates of IPV between 25–33% of ever partnered women in urban settings and up to 50% in rural areas [3, 13, 14, 15].

The first case of COVID-19 in Nepal was detected on January 13^th^, 2020. After successful quarantining of the affected patient, the second case was detected on March 17^th^, 2020, and COVID-19 began to spread quickly across the country. The first lockdown was initiated on March 23^rd^, 2020, with a surge in initial cases and then a decline, which lifted the first lockdown on July 15^th^, 2020. While the first doses of the vaccines were rolled out in January 2021, Nepal experienced a second wave from the Delta variant in April 2021, and a third wave from the Omicron variant in December 2021 and responded with intermittent lockdowns. Nearly two years later in March 2022, there were 979,140 positive cases and 11,952 deaths, with a recovery rate of 98.8% and a fatality rate of 1.2%. The COVID-related data discussed in this paper were collected in July 2020, January 2021 and July 2021.

While not yet quantified in Nepal specifically, the UN has identified exponentially rising cases of IPV across the globe since the COVID-19 pandemic [16]. Home confinement measures that increase contact with a perpetrator and reduce the ability to report violence in a safe and concealed manner are considerable causes [2, 17, 18]. This paper speculates that there are also upstream factors including economic insecurity and increased alcohol use that have been instigated by the pandemic and have contributed to the surge in VAW.

Historically in Nepal, alcohol use has been tolerable among most social groups [19]. A country-wide report from the Nepal Health Research Council in 2019 found that 23.9% of adults consumed alcohol in the past 12 months, and 20.8% within the past 12 days [20]. This survey also showed that 12.4% of males engage in heavy episodic drinking (defined as six or more drinks at any occasion in the past 30 days) compared to 1.4% of females [20].

In Nepal, and around the world, alcohol consumption and binge drinking is significantly higher among men than women with more than a quarter of men in Nepal experiencing alcohol dependence [3, 21]. Alcohol use is a predictor of aggressive and violent behavior, and women have a higher chance of experiencing sexual violence if their husband uses alcohol [6, 15, 22, 23, 24] Additionally, IPV has been found to be more frequent and severe when alcohol use is involved [3, 13].

Isolation and stress, common dynamics of the pandemic, have been correlated to rises in alcohol consumption [3, 23, 24, 25]. Since the pandemic, there has also been limited support for those suffering from an alcohol use disorder due to the overextension of healthcare systems and reduced mobility, especially in rural communities [3, 24, 25]. The increased use of alcohol as a result of the pandemic is predicted to cause a rise in IPV to correlate to the rise in alcohol use [24, 25]. Thus far, there is no evidence about how the COVID-19 pandemic has impacted alcohol use in Nepal, nor if its use has been associated with increased IPV.

The pandemic has also produced an economic downturn, which has exacerbated challenges for already vulnerable communities, especially women [11, 26]. In Nepal, 62.3% of those employed work in the informal sector which, in the wake of the pandemic, has left large portions of the population without a steady income [17]. In the 2020 VNR submitted to the UN, the Government of Nepal highlighted that the brunt of lost jobs and lost wages has been felt by women [11]. Economic insecurity at the household level has been correlated to substance abuse and various forms of conflict and IPV globally [2, 27]. A recent study, conducted with the same research team and in the same location as this study, showed that increases in IPV during the pandemic differed by level of food insecurity, a marker of economic stress [26]. Understanding if the economic downturn in Nepal has led to increases of violence can inform the impact of the COVID-19 pandemic on communities and specifically the women in these communities [26].

Women, and especially newly married women, have the lowest levels of autonomy in their household in Nepal [28]. They are highly vulnerable to the ramifications of alcohol use because they are likely to experience violence from their husband as well as their in-laws [3, 20, 26, 27]. Despite the role that household members play in IPV and the fact that IPV happens at the household level, few studies capture the perspectives of family members when seeking to understand more about causes and consequences of IPV. This paper explores pandemic-driven economic insecurity and increased alcohol use as instigators of VAW and Intimate Partner Violence (IPV) within newly married households in the rural, Nawalparasi region of Nepal. We use data from a longitudinal sample of young, newly married women in rural Nepal, combined with qualitative data collected during the pandemic with newly married women, their husbands, and mothers-in-law in the same communities.

## Data and methods

Data from this paper are from a larger study in collaboration with The Center for Research on Environment Health and Population Activities (CREHPA), Vijaya Development Resource Center (VDRC-Nepal), and the University of California San Francisco (UCSF). The primary topics of focus for the parent study were preconception nutrition, reproductive health, and women’s empowerment, with a focus on household harmony between newly married women, their husbands, and their mothers-in-law [29]. Thorough methods have been published elsewhere [29]. This paper pulls data from three phases of the study, all of which took place in the Nawalparsi district of Nepal, near the Indian border. This district was selected for its relatively low indicators of women’s status and empowerment. The multiple phases of data collection are delineated below, as well as summarized in Table 1.

**Table 1:** Phases of Qualitative and Quantitative Data Collection

Study Phase and Reference Name	Year(s)	Population	Type of study	Frequency
Phase 1: Formative Longitudinal Cohort Study	February 2017- July 2020	200 Newly married women (age 18–25)	Survey	5 rounds, every 6–9 months
Phase 2 (simultaneous): Quantitative COVID Study	January 2021	31 newly married women (age 18–25), 31 of their husbands and 31 of their mothers-in-law	Survey	Once
Phase 3 (simultaneous): Qualitative COVID Study	July 2021	15 newly married women (age 18–25), 15 of their husbands and 15 of their mothers-in-law	Qualitative interviews	Once

### Phase 1

Between 2018–2020, our binational team conducted a two and-a-half-year longitudinal study with 200 newly married women termed the Formative Longitudinal Cohort Study. The women were surveyed every 6–9 months from February 2017 to July 2020 (total of 5 rounds of data collection). Women were between the ages of 18–25 at the start of the study and had been married in the last 4 months. More details about recruitment, data collection, and initial findings are published elsewhere [29]. Based on this formative research, our team developed *Sumadhur* (meaning “Best Relationship”), an interactive group-based intervention that focused on reproductive health education, gender-based discrimination, violence prevention and nutrition education for newly married women, their husbands, and their mothers-in-law. The intervention design was informed by data from the first 4 rounds of the Formative Longitudinal Cohort Study (2017–2019). The data collection in July 2020 was an extra round added to gauge the effects of the pandemic 4 months after a nationwide lockdown and did not inform the initial development of the intervention as it was already underway. The *Sumadhur* intervention was pilot tested between 2020–2021 among newly married women and their households (total 93 participants, 31 each of newly married women, their husbands, and mothers-in-law) [30]. The newly married women in these household groups had previously been survey respondents throughout the first four rounds of the Formative Longitudinal Cohort Study. Only two women that were approached for the Formative Longitudinal Cohort Study opted to not participate in the study.

### Phase 2 (simultaneous)

Amidst the pilot study of *Sumadhur* in January 2021, another survey was designed and administered to investigate the effects of the pandemic specifically. This survey was administered to all 31 household groups that were participating in the *Sumadhur* intervention.

### Phase 3 (simultaneous)

Upon completion of the *Sumadhur* intervention, in July 2021 the local research team conducted in-depth qualitative interviews with 15 households (45 interviews total) who had participated in the intervention.

Throughout all phases of the study, participants provided written consent including consent for the in-depth interviews to be audio recorded. The consent forms were written in simple language. If the participant was illiterate, the informed consent was read aloud by the interviewers, and they were given multiple opportunities to ask questions. If they agreed to participate, their thumb print was collected in place of a signature. All surveys and interviews were held in person in a private space in participants’ homes at a time of their choosing.

The population of 200 newly married women in the Formative Longitudinal Cohort Study was the initial sample of newly married women. All subsequent phases of data collection included a newly married women who participated in this study phase, and her family unit.

### Analysis

### Qualitative

Qualitative data was analyzed using organized thematic analysis [31]. The interviews were first translated from the local language and then preliminarily reviewed to inform the development of a codebook. The codebook organized topics from the interview guide and topics that commonly arose in conversation without prompt. The question during the interviews that gave rise to this study was, “Do you think that COVID-19 has affected violence in your household or community?” The question did not directly ask participants to comment on GBV, IPV or alcohol use, rather it was open ended and allowed the themes of this paper to emerge.

A quarter of the interviews were joint-coded by a team of three (NDS, ADC, AM) in an iterative process using the software Dedoose Version 9.0.17 (2021) [32]. During this process, adjustments to the codebook were made to ensure consistency and coverage of themes. Upon completion of coding all transcripts, coded text was exported and analyzed. The present study focused on four codes relevant to the study: alcohol use, violence, IPV, and COVID-19. Qualitative data were analyzed by household to assess for patterns between family members.

#### Survey measures

**Alcohol consumption:** In Phase 1, the Formative Longitudinal Cohort Study, the husband’s alcohol consumption was measured by asking their wife “How often does your husband drink any type of alcohol?” which included the response options of rarely (< once a month), sometimes (one or two times a month), often (at least once a week), very often (everyday) or don’t know. In the last round of the Formative Longitudinal Study (3 months into the COVID-19 pandemic), an additional question was asked to the women, “Has there been any changes in your husband’s alcohol consumption in the last three months?” with the response options of yes or no.

**IPV:** In Phase 1, the Formative Longitudinal Cohort Study, IPV was measured by asking the women, “Have you faced any violence from an intimate partner?” with the possible responses of yes or no. In Phase 2, the Quantitative COVID Study, IPV was measured with the prompt, “In the last four months has your husband…” with a list of various forms of abuse from which the woman respondents could select multiple.

**COVID-19 Impact:** Questions about the impact of COVID-19 were collected in the July 2020 round of data from Phase 1, the Formative Longitudinal Cohort Study. Women were asked a question about the impact of COVID with options to select all that applied. Answer options included if there had been an increase in household conflict due to the pandemic and if someone in their household had lost income due to the pandemic.

All phases of this study received ethical approval from the University for California, San Francisco, and the Nepal Health Research Council (Reg No 385/2016).

### Quantitative

Quantitative data was cleaned by the Nepali research team and coded by the US-based team. Data was summarized and a t-test was run using STATA version 15. Exported tables were analyzed for percent change over time [33]. Graphs were developed on Microsoft Excel version 16.62 [34].

## Results

### Quantitative

**Fig. 1:**
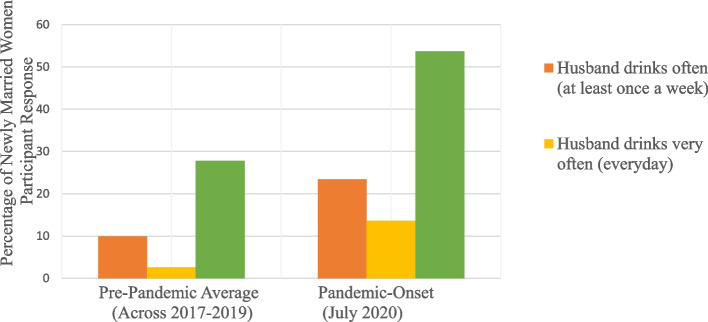
Average Pre-Pandemic Levels (2017-2019) Compared to Pandemic-Onset Levels (July 2020) of Husbands' Alcohol Use and IPV as reported by Newly-Married Women

Figure 1 shows an upward trend in reports of husbands drinking and IPV experienced by newly married women at the onset of the pandemic (round 5 of data collection for the Longitudinal Cohort Study). On average, in the pre-pandemic rounds of data collection (rounds 1-4 of the Longitudinal Cohort Study) between 2017-2019, 9.9% of newly married women said their husband drink often and 2.7% said they drink very often. Pre-pandemic, an average of 27.8% of women reported facing a form of IPV during each 6–9-month period of data collection for rounds 1-4. After the onset of the pandemic, it was found that 23.4% of women reported their husbands drank often, and 13.6% said they drank very often. In the three-month period after the pandemic-initiated lockdown in Nepal, 53.7% of women had experienced a form of IPV.

To assess the influence of the pandemic on alcohol consumption and economic insecurity, Table 2 was compiled to display the results of Round 5 of the Longitudinal Cohort Study after the onset of the pandemic in July 2020. We tested for differences in changes in household conflict and alcohol consumption between women who reported that their households lost income due to COVID-19 and those who did not report this, using t-tests. Two thirds (63.1%) of women whose household lost income reported increased conflict, compared to only about a third (36.9%) of women who did not have income loss (significantly different at the *p* < 0.001 level).

**Table 2:** Survey results of COVID-related questions in formative longitudinal cohort study round 5, July 2020

	**Has anyone in your household lost income due to the COVID-19 pandemic?**	
	**Yes**	**No**
**Due to Coronavirus, has there been any changes in household conflict among your family? (Yes)**	99 (63.06)	58 (36.94)
Has there been any changes in your husband's alcohol consumption in the past three months (women only, *N* = 102) ^***^		
** Increased**	27 (41.54)	4 (10.81)
** Decreased**	10 (15.38)	2 (5.41)
** Remained the same**	28 (43.08)	31 (83.78)

^***^ *p* < 0.001

Alcohol patterns were significantly different as well between households who lost income or not. More women who lost income reported changes in alcohol consumption, either increases or decreases. Whereas women who reported no loss in income largely remained the same in alcohol consumption. Only 10.81% of women who did not lose income reported increased alcohol use, whereas 41.54% of women who did lose income reported increased alcohol consumption.

Furthermore, 41.5% (*N* = 78/184) expressed that “not being able to meet basic needs of food and shelter” was the top challenge they were facing due to the pandemic. Thirty-three out of 184 newly married women (17.9%) ranked “worrying that someone in the family or home will use violence against me” within their top five top concerns out of 12 concerns total.

In Phase 2, the Quantitative COVID Study (in January 2021, a year into the COVID-19 pandemic), 74% (*N* = 23/31) of women reported being physically forced to have sexual intercourse with their husband when they did not want to and 68% (*N* = 21/31) reported being forced to perform sexual acts they did not want to in the past four months. Additionally, from Phase 2: the COVID Study Part 1, 42% (*N* = 13/31) of women reported that they were prohibited by their husband from getting a job, going to work, trading or earning money in the past four months.

### Qualitative

The qualitative data share personal accounts of increases in VAW in the community since the pandemic. These perspectives bring together the upstream factors of alcohol use and economic insecurity in instigating VAW. In the following quotes, it is implied that fights between family members most likely refer to violence directed towards the women in the household and could specifically be referring to instances of IPV. Below, a mother-in-law discusses the impact of the lockdown measures on family contact and aggravation.

*“During this time of Corona, people fear each other. There have been lockdowns for long periods of time. Many people don’t have a job. As all the [family] members stay at home all day, there are fights between family members for small issues. There are different types of people in the community. Some are aggressive and quarrel with family members. There has not been any kind of fights and misunderstanding in my family due to covid. But there has been violence in my neighborhood. There have been fights and quarrels among family members in their house”. (Mother-in-law #13, age 48).*

Other participants commonly discussed the financial impacts of the pandemic and what they perceived to be the resulting challenges. A mother-in-law and a newly married woman discussed how the financial struggles of being jobless led to increased rates of alcohol use and conflict in the community.

*“It’s closed everywhere. The males of the house haven’t been able to go out of the house. If there’s no job, then there’s no money. And if there’s no money, it will be hard for food and clothing. That’s why many conflicts are happening. When there’s no work to do, many kinds of thoughts come to mind. Therefore, different kinds of incidents are happening in society like theft, abuse, drinking alcohol, fighting in the society, and gambling. These kinds of incidents are heard to be happening in the society. If the person had a job to do, then they would focus on that. When they have no work, bad thoughts and opinions come to mind, because of which the society gets badly affected.” (Mother-in-law #9, age 54).*

*“Because of Covid-19, people don’t have any jobs and as they don’t have jobs it is difficult to even eat. When people don’t have enough to eat or wear, there will be fights at home, psychological distress increases, and this can be been seen in many households. My father-in-law is constantly complaining about how there is no business. When people do not have enough money to buy food, the fights and violence increases within the family”. (Newly Married Woman #14, age 20).*

Some husbands also revealed the tumultuous circumstances since the pandemic. Both quotes below identify lack of sufficient household income and a husband’s alcohol use as precursors to inflicting violence on the family. The second quote specifically refers to acts of IPV as a result.

*Due to COVID lockdown, people could not work which reduced the household income. This led to some disputes in some households of the community […]For example, the husband couldn’t go out to work so he drank alcohol in a nearby place. Upon returning home he started shouting to the family members and it created dispute.*” *(Husband #30, age 27).*

*“Yes, [in my community] there are cases of violence. This usually happens in low-income families where husbands come home drunk and start beating their wives." (Husband #9, age 26).*

*“Participant: Many people stayed home during the lockdown, they went through financial scarcity. This gave rise to violence. Due to lack of money, there were disputes leading to violence.*

Interviewer: How often was it seen?

Participant: It was frequently observed. Violence was mostly seen in the houses where husband is an alcoholic.” (Husband #13, age 22).

While not explicitly described among the quotes, a general trend identified in the qualitative data was the frequency in which each household member was likely to speak out on changes in violence since the pandemic. Mothers-in-law were the most common to say that there had been increases in violence, followed by the newly married women. Husbands were more likely to say that there had been no changes in violence since the pandemic.

## Discussion

The results illuminate an association between economic insecurity, alcohol use and VAW during the COVID-19 pandemic in Nepal. The Longitudinal Cohort survey data presented in Fig. 1 revealed that at the onset of the pandemic in 2020, there was a simultaneous increase in newly married women’s reports of their husbands’ alcohol use and their reports of experiencing IPV. The increase in reports of IPV compared to pre-pandemic averages suggests that the conditions of the pandemic, including increased economic stressors, isolation and overextended healthcare systems were contributing to a spike in IPV. The t-test conducted from round 5 of The Cohort (Table 2) indicated that loosing household income correlated with increased household conflict and more dramatic changes in alcohol consumption (majority being an increase in alcohol consumption). These associations were significantly different than newly married women (*p* < 0.001) who did not have income loss and mostly reported no changes in household conflict or in alcohol consumption. Additionally, fears of not being able to meet basic needs of food and shelter and experiencing violence were top concerns of newly married women in July of 2020. The data is consistent with other studies that show a direct correlation between IPV and alcohol use or economic hardship [3, 13, 22, 35, 36].

The Quantitative COVID Study in January 2021 found up to 74% of women had experiences of IPV, whereas the rate found in July 2020 in The Cohort Study was 53.7% of women (Fig. 1). This suggests climbing rates of IPV as the pandemic and its stressors persisted. In this round of data collection, there was also a high percentage of women (42%) that reported being prohibited from getting a job or trading to earn money. This reiterates the lack of autonomy that women have in their households and suggests that gender barriers have compounded the economic stressors that have led to cascades of violence [17, 28].

The qualitative data give voice to the household unit and show the community and family level impact of the pandemic. While not specifically prompted to discuss alcohol use or economic insecurity, these were reiterated themes when participants were asked if the pandemic had affected violence in their community. The lockdowns led to close confinement with family members and alcohol use was said to instigate violence, and specifically IPV in some cases. Additionally, it was observed that mothers-in-law and newly married women were more likely to speak out on changes in alcohol use and violence, and husbands were the least likely to report these increases. These reports highlight the frequency of mothers-in-law and newly married women as subjects of violence and support global trends of higher alcohol use among men than women, and higher rates of violence against women than men [21, 35].

Together, these results align with previous findings that alcohol consumption rises during times of crisis, uncertainty, and economic stress [2, 24, 25, 35]. They echo the vulnerability of women during these times, and the increased risk of VAW and IPV when alcohol is involved [13, 22, 35] These findings elaborate on studies that show women who did not have a steady or sufficient income of their own reported higher rates of IPV than women who did have adequate and steady employment, as being financially insecure can increase vulnerability to violence [22]. Our data underscore the critical need for efforts to address these intersecting factors and prevent placing women at the apex of harm during times of crisis.

Beyond the findings of this study, successful interventions in similar settings in Nepal suggests multiple avenues forward to prevent VAW. The implementation of interactive, group-based interventions like *Sumadhur*, could increase community and household-level knowledge surrounding gender equality, VAW and IPV prevention and country-specific laws on the safety and protection of women [29]. Parallel interventions targeting husbands and fathers-in-law could specifically address alcohol use as a tangential method of decreasing rates of VAW and IPV [3, 19, 21, 28, 35, 36]. Additionally, there are studies showing that increased access to hotlines, reporting services and healthcare for victims of violence may be necessary in rural and under resourced areas, especially during times of increased hardship [1].

In Nepal, targeting persistent and constrictive gender norms is another important gateway for change. It is important that program intervention especially for young newly married couples address the wider societal basis of gender-based discrimination and violence. Educating on an equal worldview within programs designed to address the self-image of newly married women, their own expectations of their role as well as expectations of their spouses, families, and children, could be greatly beneficial.

## Limitations

Our data is unique in that it collects longitudinal data both prior to and during the COVID-19 pandemic, giving insight into how the lives of people in the Nawalparasi district of Nepal changed due to the pandemic. It combines longitudinal data with triadic qualitative data, adding richness and multiple voices to our understanding of the interplay between COVID-19, alcohol use, and IPV. Despite these strengths, this study has four main limitations. Data was collected from one district of Nepal, thus limiting the generalizability of the findings to other parts of Nepal or other countries. Not all participants were involved in every phase of data collection, and some of the participants from Phase 1: The Longitudinal Cohort Study did not participate in Phases 2 and 3 of data collection as they were a subset of this cohort. Additionally, there were logistical disruptions in data collection due to the pandemic, and some of the quantitative and qualitative rounds of data were collected up to one year apart (between July 2020-July 2021). Thus, there may be more differences between the experiences in the two samples, although they were both newly married women (and their households) and were from the same district of Nepal.

Alcohol use, VAW and IPV were not the main focus of the parent study, and thus we are limited in the detail of the data about these experiences. Additional future research focusing on causes and levels of increased alcohol consumption, the link to VAW and IPV, and interventions that can reduce these forms of violence in times of social crisis, such as COVID-19 is urgently needed.

## Conclusion

Progress that has been made on gender equality and VAW prevention in the past few decades has been threatened by the COVID-19 pandemic. In Nepal, alcohol use and economic insecurity has increased, with rates of household conflict and IPV simultaneously on the rise. The findings from this study underscore urges from the UN that women should be at the center of recovery efforts from the pandemic [37]. Community-based capacity building through women’s empowerment initiatives and policy to protect the safety of women should be high priority to reach the UN SDGs 5 and 16 and to create healthier, safer, and more equitable societies in Nepal and across the globe. 

## Data Availability

The datasets used and/or analyzed during the current study are available from the corresponding author on reasonable request.
